# Rabies Risk: Difficulties Encountered during Management of Grouped Cases of Bat Bites in 2 Isolated Villages in French Guiana

**DOI:** 10.1371/journal.pntd.0002258

**Published:** 2013-06-27

**Authors:** Franck Berger, Noëlle Desplanches, Sylvie Baillargeaux, Michel Joubert, Manuelle Miller, Florence Ribadeau-Dumas, André Spiegel, Hervé Bourhy

**Affiliations:** 1 Institut Pasteur de la Guyane, Cayenne, French Guiana; 2 Centre Hospitalier de Cayenne, Cayenne, French Guiana; 3 Direction de l'Alimentation, de l'Agriculture et de la Forêt de Guyane, Cayenne, French Guiana; 4 Institut Pasteur, Centre National de Référence de la Rage, Paris, France; The Global Alliance for Rabies Control, United States of America

## Abstract

In French Guiana, from 1984 to 2011, 14 animal rabies cases and 1 human rabies case (2008) were diagnosed. In January 2011, vampire-bat attacks occurred in 2 isolated villages. In mid-January, a medical team from the Cayenne Centre for Anti-Rabies Treatment visited the sites to manage individuals potentially exposed to rabies and, in April, an anti-rabies vaccination campaign for dogs was conducted. Twenty individuals were bitten by bats in 1 month, most frequently on the feet. The median time to start management was 15 days. The complete Zagreb vaccination protocol (2 doses on day 0 and 1 dose on days 7 and 21) was administered to 16 patients, 12 also received specific immunoglobulins. The antibody titration was obtained for 12 patients (different from those who received immunoglobulins). The antibody titers were ≥0.5 EU/mL for all of them. The serology has not been implemented for the 12 patients who received immunoglobulins. Accidental destruction of a vampire-bat colony could be responsible for the attacks. The isolation and absence of sensitization of the populations were the main explanations for the management difficulties encountered. Sensitization programs should be conducted regularly.

## Author Summary

Rabies is a disease almost invariably fatal in humans once the first clinical signs appear. In French Guiana bats represent the virus reservoir, especially vampire bats. From 1984 to 2011, 14 animal rabies cases and 1 human rabies case (2008) were diagnosed. In case of bat bite, anti-rabies immunoglobulins (RIG) and rabies vaccine must be rapidly administrated. The specific rabies management is exclusively performed by Centre for Anti-Rabies Treatment (CART), located at the Institut Pasteur in Cayenne, the prefecture of French Guiana, and 6 Anti-Rabies Treatment Outposts distributed along the coastal edge and along the two main rivers. Only a CART physician can administer RIG. In January 2011, vampire-bat attacks occurred in 2 isolated villages. In mid-January, a medical team from the CART visited the sites to manage individuals potentially exposed to rabies and, in April, an anti-rabies vaccination campaign for dogs was conducted. The most relevant contribution of this study is to underline difficulties to provide rabies post-exposure prophylaxis to remote populations exposed to bat rabies in the Amazonian region and to show the lack of awareness of these rural populations concerning rabies and the risk associated to vampire bats.

## Introduction

A deadly viral zoonosis distributed worldwide, rabies is responsible for about 55,000 deaths annually, mainly in Asia and Africa [1]. Several viruses, all belonging the *Rhabdoviridae* family, cause rabies, with 12 species from the genus *Lyssavirus* having been described [2]. The lyssaviruses circulating in the Americas are all rabies virus (RABV) species [3]–[5].

In 2004, for the first time in Latin America, more human rabies cases were transmitted by vampire bats than dogs [6]. In Brazil, which abuts French Guiana, 10–30 cases were declared annually from 1995 to 2004 [7] and, in 2005, the majority of the 60 human rabies cases transmitted by bats notified in Latin America occurred in northern Brazil, notably in Pará and Maranhão states, which are close to French Guiana [8], [9]. In Suriname, northwest of French Guiana, the last human desmodin-type rabies epidemic was reported in 1975, and a bat-transmitted case was declared in 1998 [6].

In French Guiana, a French overseas department that is located in South America, from 1984 to 2011, 14 rabies cases were diagnosed in animals, including 2 dogs and a cat. The principle of surveillance of rabies in French Guyana is based on a network of veterinarians and is similar to what is in place in the other parts of France [10]. All were caused by RABV variants associated with vampire bats (mainly *Desmodus rotundus*). In May 2008, the first autochthonous human rabies case was diagnosed in France since 1924 [11], [12]. The transmission's origin could not be identified, but virus-typing results indicated that it was a desmodin-type rabies virus [13]. In 2009, a desmodin-type rabies virus was detected in a fruit-eating bat captured in Remire-Montjoly, near Cayenne [13].

**Figure 1:**
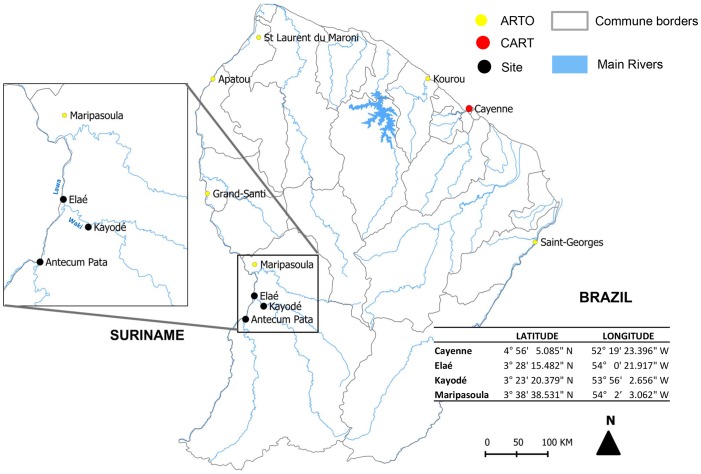
Locations of the different points of interest. Locations of the Centre for Anti-Rabies Treatment (CART), Anti-Rabies Treatment Outposts (ARTO), and Elaé and Kayodé villages.

Rabies, once the clinical signs appear, is, with rare exceptions, fatal for humans. However, it is possible to prevent symptom onset by vaccination after exposure to the infectious agent. Rabies post-exposure prophylaxis (Rabies PEP), including nonspecific measures (antibiotherapy and tetanos prevention), consists of washing the wound and vaccination associated, when necessary, with specific anti-rabies immunoglobulins (RIG) [14]. In French Guiana, the prevention and control of rabies follow French regulations. The Pasteur rabies vaccine (purified Vero cell vaccine) is used and either the Zagreb (2 doses on day 0, followed by 1 dose on days 7 and 21) or the Essen protocol (1 dose on days 0, 3, 7, 14 and 28) [13] can be applied. Human RIG (Imogam rabies 150 IU/mL) are infiltrate as much as possible by injection in the wound, with the rest of the solution given intramuscularly. The dose is proportional to the patient's weight (20 IU/kg). Rabies PEP is only administered in Health Ministry-approved centres [15] or their outposts and RIG can only be given in the Centre for Anti-Rabies Treatment (CART) [10], [16]. Two hospitals, one surgical-medical centre and 21 health centres distributed throughout the country participate to the general care of rabies exposed patients. The specific rabies management is exclusively performed by CART, located at the Institut Pasteur in Cayenne, the prefecture of French Guiana, and 6 Anti-Rabies Treatment Outposts (ARTO): the hospital of Saint Laurent-du-Maroni, the surgical-medical centre of Kourou and 4 health centres (Maripasoula, Grand Santi, Apatou and Saint Georges de l'Oyapock) (Figure 1). Since 2008, each year, between 350 and 450 patients have consulted for potential rabies exposure. In 2011, 451 patients have consulted and bats were involved for 106 patients [17]. However, the huge size of the territory (84,000 km^2^) mainly covered by Amazonian forest and the poor road network makes accessibility to treatment difficult for part of the population.

In French Guiana, preventive (preexposure) vaccination for professionals at risk and for travelers to zones of high enzootic canine rabies, particularly young children, followed WHO guidelines (1 dose of vaccine per the IM route on days 0, 7, 21 or 28). In the case of continued exposure, serological monitoring may be indicated, with vaccine boosters as needed [14], [18].

For animals, following the 2008 recommendations of the French agency for public health and food safety [19], a ministerial decree [20], [21] made anti-rabies vaccination of domestic carnivores (dogs, cats and ferrets), cattle, horses, sheep and goats mandatory in French Guiana, with Direction of food, agriculture and forests (DAAF) technicians administering vaccinations to livestock [22].

### Context

Wednesday, 5 January 2011, a Maripasoula (Figure 1) Centre for Prevention and Care (CPC) doctor notified CART that 8 patients from the village of Elaé, located in southwest French Guiana, had been bitten by vampire bats during the night of 24–25 December. They were not vaccinated against rabies and should receive immune therapy at CART in Cayenne. To do so, they had to reach Maripasoula, located about 1 h away by motorized pirogue, and then take a 1-h flight to Cayenne. Because some patients refused to go to Cayenne and the lack of available seats on a plane, a CART physician was dispatched to Elaé on 7 January by helicopter. Vaccines and RIG were transported into a cool box with ice-packs.

By Tuesday, 11 January, 7 more individuals were reported being bitten by bats, all from Kayodé. This village of 150 inhabitants is located 1 h by pirogue southeast of Elaé (Figure 1). A second medical team was flown to the Maripasoula CPC on 14 January to care for these new victims and those not seen during the first visit. Because inhabitants of the 2 villages could not be contacted by telephone or internet, on 12 January, a Maripasoula CPC physician going to Antecum Pata stopped in both villages to inform the bite victims of the arrival of the medical team on the 14 January. Victims were managed on 7 and 14 January by a CART physician assisted by an EMS doctor to anesthetize the patients with nitrous oxide (Kalinox). The presence of an EMS doctor is a common practice in France mainly because of the extremely painful RIG injections into the extremities (fingers, toes) especially in children <10 years old. In agreement with the DAAF and the French national agency for public health, food, environment and occupational safety (ANSES), it was decided to vaccinate all the dogs and cats present in the 2 villages and to conduct an inquiry on the canine population.

The objectives of this study were to describe: 1) the outbreak of vampire-bat bites in 2 Amerindian villages in French Guiana, 2) the management of bite victims and the associated difficulties, 3) the inquiry conducted on-site, and 4) the measures established and those proposed to improve rabies prevention in isolated communes.

## Materials and Methods

This descriptive study concerned the vampire-bat-bitten patients from the villages of Elaé and Kayodé who consulted at the Maripasoula CPC in December 2010 and January 2011. Information about the environment, preventive methods used, history of bat bites, knowledge of rabies and means of prevention was collected from the patients during their treatment management. For the patients <10 years, informations related to knowledge of rabies and means of prevention were not collected, being considered as unreliable.

During the campaign to vaccinate dogs, a standardized questionnaire (Text S1) was completed with the owner.

In accordance with French practices, serology testing was implemented when protocol violations of the post exposure regimens was reported. When observed, anti-rabies-antibody titers were determined for all vaccinated patients to evaluate postvaccinal immunogenicity. Platelia Rabies II (Bio-Rad, Marnes-La Coquette, France) was used to detect anti-rabies-glycoprotein antibodies [23]. Serum was preferentially tested as of the 14^th^ day after the last dose; a titer ≥0.5 equivalent units (EU)/mL means the patient requires no further vaccine [14]. When anti-rabies antibodies were tested simultaneously with vaccination, the interval between the titer and the last vaccine dose took into account the date of the previous dose, considered the “last active dose”.

Data were entered and analyzed with Epi-info v3.5.1 software (Centres for Disease Control and Prevention, Atlanta, GA). Attack rates were compared with a χ^2^ test.

A previous “normal declaration” to the CNIL (Commission Nationale de l'Informatique et des Libertés) submitted by the CART of French Guyana in 2008 and accepted, allows the physician of the CART to collect medical data of patient exposed to rabies. So, it was not necessary to contact an ethical committee in order to investigate these patients.

Regarding the animal owner survey, questionnaires were administered by the CART physician at the same time as dogs vaccination by veterinarians of the DAAF (Direction de l'Alimentation, de l'Agriculture et de la Forêt en Guyane). This survey was performed under a health sanitary mission organised by French authorities (DAAF). In France, when such a public health mission is organised, it's not necessary to provide an information note to the people that are investigated. However, for this investigation, owners were orally informed by the CART physician in charge.

Therefore, no ethical approval was necessary. Furthermore, evaluation of vaccinal policy and monitoring of antibody response in patients exposed to rabies and receiving post-exposure prophylaxis is part of the missions of the National Reference Centre for Rabies in Institut Pasteur [24].

## Results

### Description of the cases

Twenty patients were bitten by vampire bats during the night: 8 (40%) at Elaé and 12 at Kayodé, representing an 8% attack rate (respectively, 8/100 and 12/150, p = 1.0).

**Figure 2:**
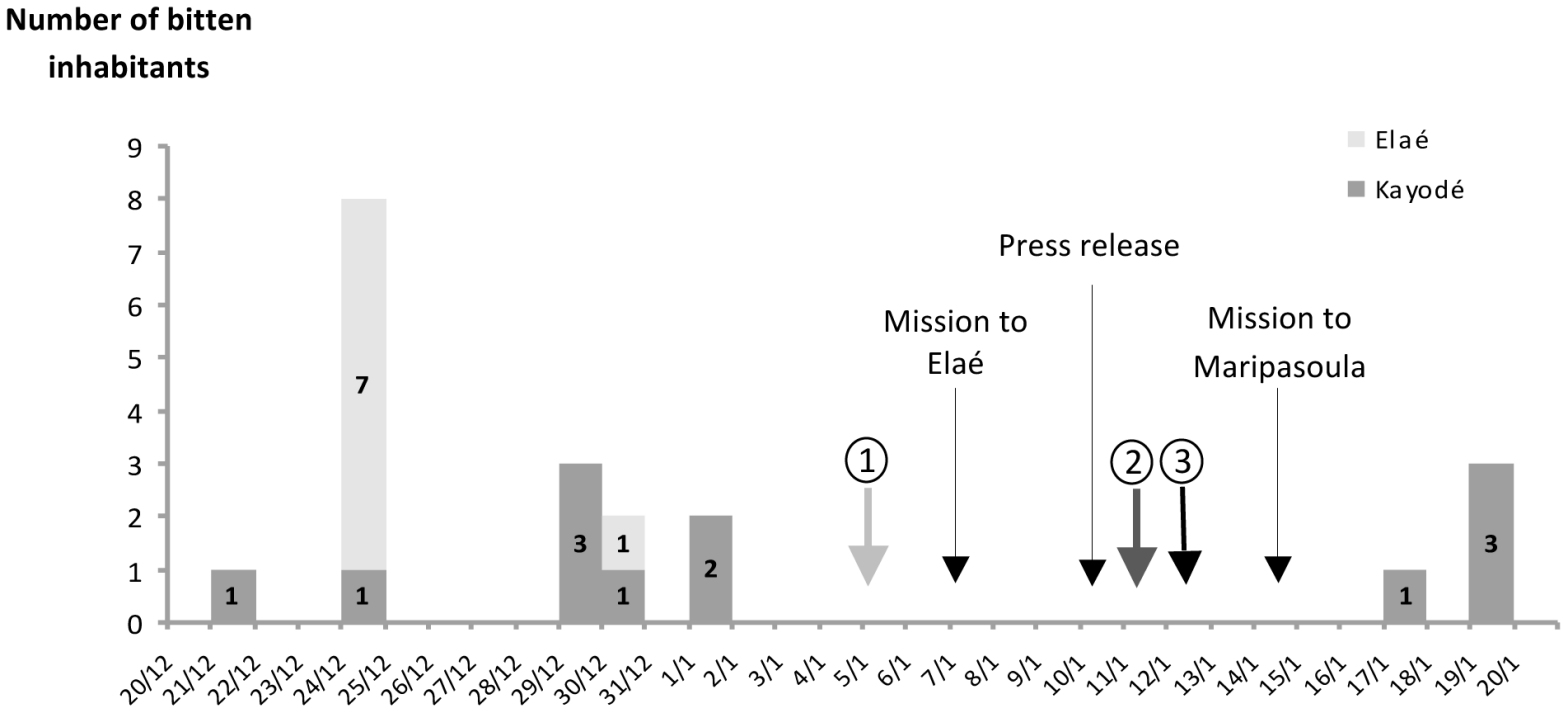
Epidemic curve. Outbreak clusterings of bat-bitten inhabitants in Elaé and Kayodé in December 2010 and January 2011. 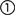 First notification to CART of bat bites 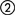 Second notification. 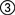 Elaé and Kayodé bat-bitten inhabitants informed of the Centre for Anti-Rabies Treatment physicians' intervention on 14 January at the Centre for Prevention and Care of Maripasoula.

The outbreak clustering showed a peak of bat attacks at Elaé the 24 December (*n* = 7) (Figure 2). Patients belonged to the same family and lived in the same house (except patient 8). Two attack periods occurred at Kayodé: the first between 21 December and 1 January (*n* = 8) and the second from 17 to 19 January (*n* = 4). The 12 Kayodé victims belonged to 5 families living separately.

The 20 patients, 45% males, had a median age of 13.7 [range: 0.7–36.0] years. The bite site was not specified for 2 victims, but the feet (toes, heel) were the most frequent (12/18, 67%), followed by the hands (8/18, 44%) and face (eyebrow arch, nose: 2/18, 11%). Patients had a median of 2 [range: 1–4] bites.

### Management

**Figure 3:**
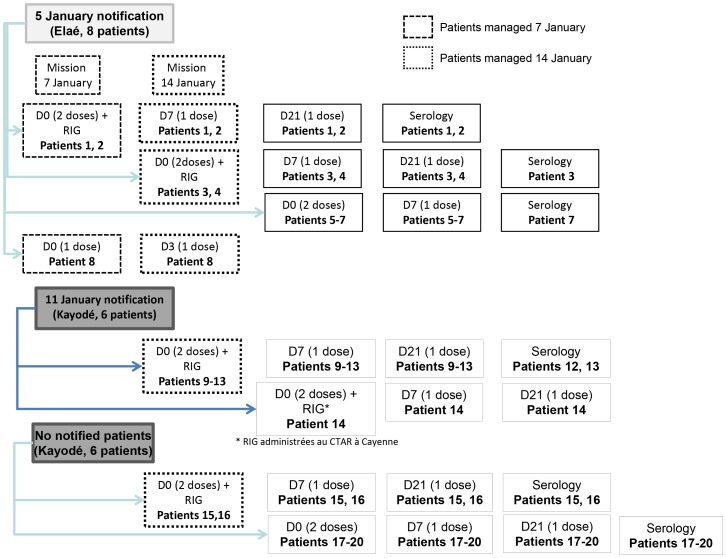
Management of the Elaé/Kayodé bat-bitten inhabitants. Management of the Elaé/Kayodé bat-bitten inhabitants in December 2010 and January 2011. *Anti-rabies immunoglobulins administered at the Cayenne Centre for Anti-Rabies Treatment.

By Friday, 14 January, 14 vampire-bat–bitten victims were counted, 8 at Elaé and 6 at Kayodé (Figure 3, patients 1–14). During the medical team's visit on 7 January, only 3 of the listed patients could be treated. During the 14 January visit, 2 new bat-bite victims from Kayodé consulted (patients 15 and 16). In total, 12 of these 16 (75%) patients consulted at the Maripasoula CPC on 14 January (patients 1–4, 8–13, 15 and 16) (Figure 3). During those 2 medical visits, 50 RIG doses and 44 vaccine doses were brought to the CPC and 29 (58%) and 23 (52%, respectively, were administered. After CART physicians left, 4 new Kayodé victims consulted for 1 or several bat bites (patients 17–20).

**Figure 4:**
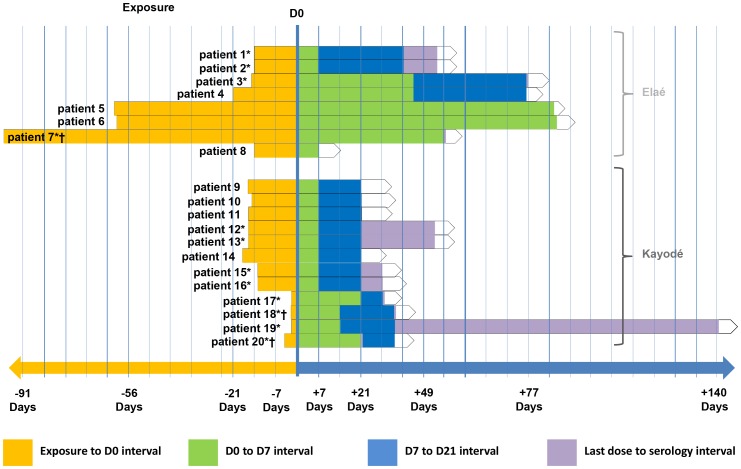
Distribution of the patients according to the interval between exposure and treatment. Distribution of the 20 patients according to the intervals between exposure and D0 (beginning of postexposure prophylaxis), D0 and D7, D7 and D21, and last active dose and serology (Elaé, Kayodé, December 2010–June 2011). * Patients with anti-rabies–antibody titers. † Serology and vaccination at the same time.

The median time to start management was 15 [range: 2–96] days (Figure 4; Table 1). None of the victims had previously been vaccinated against rabies. Among the 20 patients exposed, all started anti-rabies vaccination according to the Zagreb protocol, 16 (80%) received the complete 4-dose regimen; three received 3 doses (patients 5–7) and patient 8 received only 1 dose. The median time to the administration of the third dose (day 7) was 7 [range: 7–85] days. The median time to the administrations of the first and fourth doses (normally days 0 and 21) was 24.5 [range: 21–75] days. Twelve (60%) patients received RIG: 9 at Maripasoula, 2 at Elaé and 1 at CART in Cayenne (patient 14). For each of them, RIG were administered at the same times as the first 2 vaccine doses, corresponding to protocol day 0.

**Table 1:** Specific medical care of the 20 patients bitten by vampire bats (Elaé, 1 Kayodé, December 2010–2 January 2011).

Patient number	Village*	Age	Sex	Exposure date	Vaccination dates			RIG dose ** (IU)	Serology	
					J0 (exposure-J0 delay)	J7 (J0-J7 delay)	J21 (J0-J21 delay)		Date (delay***)	Titers # (EU/mL)
1†	E	8.6	F	24/12	07/01 (14)	14/01 (7)	11/02 (35)	440	22/02 (11)	2.5
2†	E	9.9	F	24/12	07/01 (14)	14/01 (7)	11/02 (35)	360	22/02 (11)	3.1
3†	E	1.1	M	30/12	14/01 (15)	21/02 (38)	30/03 (75)	180	01/04 (2)	>4.0
4†	E	16.9	F	24/12	14/01 (21)	21/02 (38)	30/03 (75)	1200	ND	ND
5†	E	2.4	M	24/12	22/02 (60)	17/05 (85)	ND	ND	ND	ND
6†	E	28.0	M	24/12	21/02 (59)	17/05 (85)	ND	R	ND	ND
7†	E	2.4	F	24/12	30/03 (96)	17/05 (48)	ND	ND	17/05 (48)	2.3
8	E	31.0	M	24/12	07/01 (14)	14/01 (7)	ND	R	ND	ND
9‡	K	36.0	M	29/12	14/01 (16)	21/01 (7)	04/02 (21)	1980	ND	ND
10‡	K	10.3	F	30/12	14/01 (15)	21/01 (7)	04/02 (21)	1060	ND	ND
11‡	K	5.6	F	29/12	14/01 (16)	21/01 (7)	04/02 (21)	380	ND	ND
12‡	K	15.0	M	29/12	14/01 (16)	21/01 (7)	04/02 (21)	980	28/02 (24)	1.9
13‡	K	12.4	F	21/12	14/01 (24)	21/01 (7)	04/02 (21)	1200	28/02 (24)	0.5
14‡	K	18.2	M	01/01	19/01 (18)	26/01 (7)	09/02 (21)	1260	ND	ND
15‡‡	K	20.9	F	01/01	14/01 (13)	21/01 (7)	04/02 (21)	1240	11/02 (7)	1.7
16	K	24.2	M	01/01	14/01 (13)	21/01 (7)	04/02 (21)	1260	11/02 (7)	2.6
17†	K	6.2	F	19/01	21/01 (2)	11/02 (21)	18/02 (28)	R	19/02 (1)	>4.0
18††	K	0.7	F	19/01	21/01 (2)	04/02 (14)	22/02 (32)	R	22/02 (18)	3.9
19††	K	34.6	F	19/01	21/01 (2)	04/02 (14)	22/02 (32)	R	08/06 (106)	>4.0
20‡‡	K	27.2	M	17/01	21/01 (4)	11/02 (21)	22/02 (32)	R	11/02 (21)	0.9

† «I» family,

‡ «O» family,

†† «B» family,

‡‡ «C» family,

* E = Elaé, C = Kayodé;

** anti-rabies immunoglobulins (R = refusal, ND = not done);

*** running delay between serology and the last vaccine injection excluding injection carried out the same day that the serology;

# : enzyme-linked immunosorbent assay (ELISA) method.

Because of protocol violations, serology was requested for all the patients but only 12 (60%) patients went to the CPC serology control (Figures 3 and 4; Table 1). The median time between the last active dose and serology was 14.5 [range: 1–106] days. All 12 victims had titers ≥0.5 EU/mL. The antibody titer for patient 13, a 12.4 year old who had received the complete Zagreb protocol, was lower than the others (0.5 EU/mL) when tested 24 days after the last dose (Table 1). As for patient 13, serology was run after day 21 for 9 patients; the interval between the last dose and testing exceeded 14 days for patients 12, 13 and 19, and was shorter for patients 1–3 and 15–17 [range 1–11] (Table 1). All patients were in good health 1 year after starting PEP.

### Investigations

**Figure 5:**
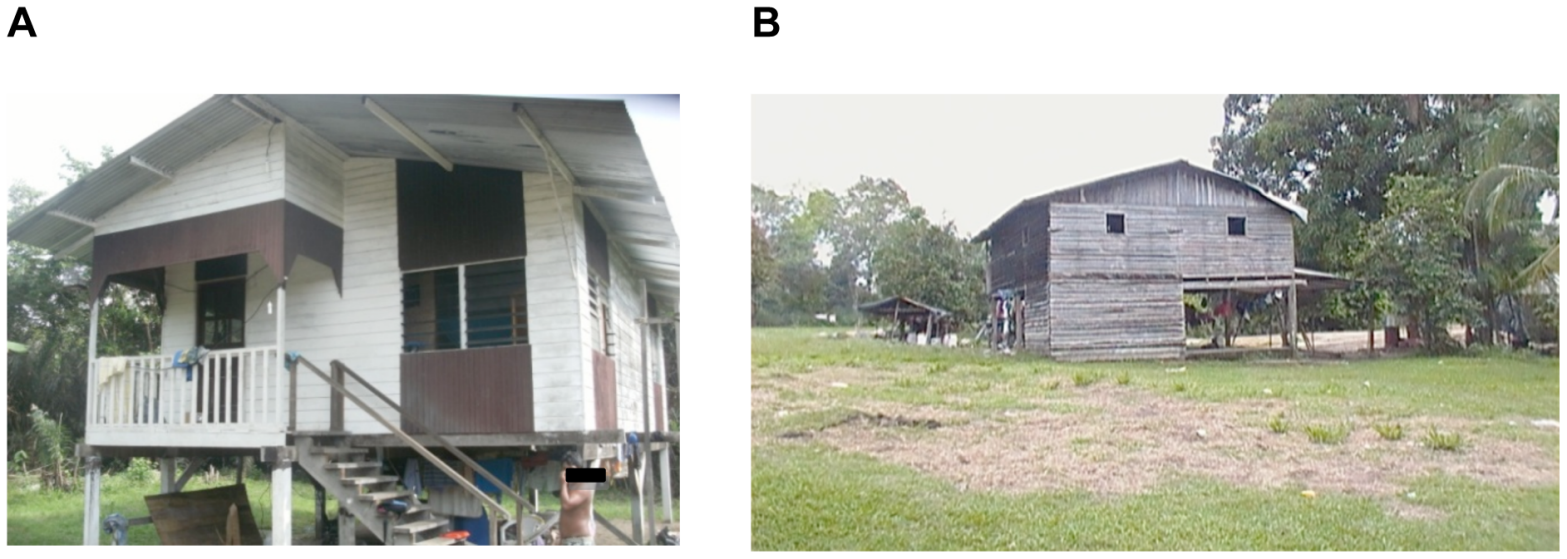
Typical local wood houses. Typical wood houses: (A) where 7 Elaé inhabitants were bitten by bats, and (B) another and its environment, showing the proximity of the forest.

At Elaé, the house (Figure 5A) in which the majority of bite victims slept was located in the village and had numerous openings that would allow bats to enter. At Elaé and Kayodé, most homes are made of wood and situated close to the forest (Figure 5B). Among the 9 patients responding, all lived in wood houses on stilts and declared sleeping with mosquito nets every night. Patient 4 declared having been bitten just once by a bat during the last 5 years but had not received PEP. Among 7 patients >10 years old, only 1 (14%) had previously heard of rabies and 3 (43%) knew the preventive measures to avoid bat bites (netting over the openings, mosquito nets around beds and light).

Kayodé patients reported that, in mid-December, they had cleared an area of forest on the village periphery destined for planting crops.

### Communications

After the return of the 7 January medical team, the Regional Health Agency (RHA) issued a press release on 10 January about the vaccination mission (Text S2) and the Prefecture of French Guiana issued another on rabies and its preventive measures (Text S3).

During the second medical visit on 14 January, the RHA and DAAF provided posters and information brochures on rabies in French Guiana and what should be done in the case of a dog bite or scratch. Ample mosquito nets were distributed to village inhabitants.

### Veterinary inquiry

Thirty-four dogs were counted in the 2 villages, for a man/dog ratio of 8.8. Eight families with 16 dogs, i.e., 47% of the dogs present at the 2 sites (11 in Elaé and 5 at Kayodé), could be questioned. Two families had 4 dogs, 2 had 2 dogs and 4 families had 1 dog each. The median number of dogs per family was 1.5. At Elaé and Kayodé, dogs were rarely attached. Properties are not fenced, leaving the animals the possibility to roam free. Most dogs (12/16, 75%) were cared for by women. Only the dog owners fed their dogs (not the neighbors), primarily with leftovers of family meals. All family members could be in contact with the animal.

Seventy-five percent of dogs are trained to hunt; the other 25% are guard dogs. None of the dogs was considered a pet and none had been vaccinated against rabies nor identified. All the dogs (except 1 at Kayodé that ran away) were vaccinated after electronic identification. In all, 17 dogs and 3 cats were vaccinated in Kayodé and 16 dogs in Elaé.

During the previous 2 months, 1 vampire-bat bite was reported by the owners of 7/16 (44%) dogs in Elaé and in Kayodé. At Elaé, 1 dog was bitten 3 times. In 2010, 1 family in Elaé and another in Kayodé reported the deaths, all of unknown causes, of 3 and 5 dogs, respectively. In addition, the Kayodé patients seen on 14 January reported the death of 1 of the dogs in the days following a bat bite on the muzzle in December 2010.

## Discussion

The most relevant contribution of this study is to underline difficulties to take in charge isolated populations exposed to bat rabies in the Amazonian region and to highlight the lack of knowledge of rabies among this Amerindian population. The episode related here described clustered vampire bat bites and potential rabies exposures in two remote small villages in French Guiana from December 2010 to January 2011. The attack rate observed (8%) with a predominance in young females bitten on the feet are in agreement with previously reported findings in similar settings. In September 2005, during a blackout in a village in northeast Brazil (Maranhão State), 57 (8%) of the 685 inhabitants had been bitten by bats [25]. However cross-sectional studies realized in Brazil [26] and Peru [27] showed that more than a third of the local population had been exposed to bat bites. Another study described rabies outbreaks that occurred in May 2004 in northern Brazil (Para State), and the feet were also the primary bat-bite site [28]. In September 2005, the epidemiological inquiry conducted in Brazil showed that youths <17 years old represented a population at risk because, according to those authors, they had a greater tendency to uncover their extremities during their sleep. During the inquiry conducted in French Guiana, all the patients questioned said that they slept under a mosquito net, thereby confirming that it is, nonetheless, possible to be bitten if the limb is in contact with or extends beyond the netting. The coincidence with Christmas celebration is noticeable, this period might played a role in increasing the opportunities for exposures among the population.

Gilbert et al. [27] reported a possible seasonal incidence of rabies virus infections from vampire bats to humans and cattle occurring shortly after the onset of the rainy season. Data from the Cayenne CART [29] showed that exposure to vampire bats mostly occurred in the forest or its proximity, with a peak during the dry season (mid-July to mid-November), which is more suitable to outdoor activities (crop cultivation, hunting, fishing, hobbies). The presence of livestock [30], the characteristics of the terrain (proximity of a river, forest, crop cultivation) [31] are factors associated with the presence of vampire bats. No animal husbandry is carried out in Elaé/Kayodé, but the clearance of a forested zone in December near Kayodé might have been responsible for this outbreak. It had been shown in Brazil that deforestation, by favoring the contact between humans and bats, was associated with human desmodin-type rabies outbreaks [6], [32]. Vampire bats generally colonize poorly lit sites, like caves or hollow tree trunks. The destruction of the colony site could have provoked the dispersion of the colony and the bats in search of a new location to colonize might have bitten the Elaé and Kayodé inhabitants, despite the distance separating the 2 villages. Vampire bats can cover several dozen kilometers in the search for food [33].

Because of the difficulties to put in place such captures (necessity to spend several days on the site with material and staff without certainty to capture some bats), no vampire bats captures were organized at Elaé/Kayodé. A preliminary study on the carriage of the rabies virus by bats (hematophagous, frugivorous and insectivorous) in French Guiana showed that <0.1% of bats captured since 2009 were carriers (saliva and blood) (Anne Lavergne, personal communication) with a rabies seroprevalence of 6.6% for *Desmodus rotundus*. Serological studies conducted in São Paulo, Brazil [34] and Peru [35] found seroprevalence rates of 1–40%, depending on the species and the type of habitat. *Desmodus rotundus* rabies seroprevalence was 12% in Brazilian study and 14% in those conducted in Peru.

At Elaé/Kayodé, the bat attacks of humans seem to have been uncommon, since only 1 of the 9 questioned patients declared having previously been bitten by a bat. This rate was much lower than those found during human rabies outbreak in Maranhão (Brazil) in 2005, when 42% of the local population had previously been bitten by bats [6]. Nevertheless, the presence of vampire bats in the area around the 2 villages is strongly suspected because several of their dogs had previously been bitten. Moreover, in 2010, 3 dogs in Elaé and 5 in Kayodé had died; the clinical picture could not be described and, unfortunately, the tissue samples to be taken when rabies is suspected had not been obtained.

Management of exposure to the risk of rabies must be undertaken rapidly. To maximize the ability to manage all the exposed patients, considerable efforts were put in place including dispatch of medical teams from Cayenne to Elae and Maripasoula by helicopter and plane, respectively. However, the median time to available care of the patients in the 2 villages was 15 days, with 1 patient seen 96 days after exposure. The prolonged delays can be explained by 1) the difficulty of contacting the Amerindian population because of the lack of modern means of communication (telephones, internet) and because of their frequent displacement to fish, hunt, farm; 2) the absence of sensitization to the risks linked to rabies ; and 3) the logistical constraints for health officials to reach distant villages that do not have pirogues so they have to be rented locally. It should be noted that the initial notification of the first 7 bite victims was fortuitous because it was during the systematic public health visit to Elaé on 5 January that the Maripasoula CPC physician observed that several children had homemade bandages on their limbs. On asking about them, he learned of bat bites dating from 25 December (i.e., 11 days earlier) and informed Institut Pasteur in French Guiana upon his return.

Many protocol violations were reported during these 2 episodes. Only 12 of the 20 patients received the complete vaccination protocol combined with RIG and for 4 of them, the interval between doses was not respected. During the 2 medical team visits, only 1 patient refused RIG injection. On the other hand, all the patients that had consulted at the CPC after 14 January refused RIG. For the latter, the need to go to Cayenne was the reason for the refusal, despite the hospital offer of paid transportation and treatment. The 12 exposed patients tested had anti-rabies-antibody titers ≥0.5 EU/mL. The serology used an ELISA as it was the only method available locally to quickly obtain patients' antirabies titers. Furthermore, all 20 patients were still alive one year later. That finding is reassuring, given 1) the vaccination-protocol violation for 67% of the patients, 2 of whom received only 2 doses (day 0) at the time of blood sampling; and 2) the risks of low anti-rabies-antibody titers because of some short intervals between the last dose received and blood sampling (median interval of 14.5 days) [36]–[38].

The literature review by Schneider et al [6] showed that, during outbreaks of human desmodin-type rabies, the distance between place of residence and health facilities represented a risk factor as did the lack of knowledge concerning rabies and the role of bats. That was also true for the villages of Elaé/Kayodé, located several hours from Cayenne (the only site authorized to administer anti-rabies immunotherapy), and their inhabitants seemed poorly informed, as only 1 declared knowing about rabies and none was aware of the rabies-caused death of a French Guianan in May 2008, despite the intense media coverage at the time.

The vaccination of domestic animals is one of the preventive measures against bat rabies; it is obligatory in French Guiana [20] but rarely followed in rural villages as shown by the veterinary inquiry. As a matter of fact, this measure is hardly applicable as the closest veterinarian susceptible of identifying and vaccinated land carnivores is at Saint-Laurent-du-Maroni, about 2 days by pirogue from Maripasoula.

### Conclusions and recommendations

The remoteness of populations living in villages far from health facilities, and the isolation of these populations because of the lack of modern means of communications represent major obstacles for medical management. These difficulties are even more dramatic when the inhabitants are unaware or poorly aware of the risk concerning rabies, as for these two Amerindian populations of French Guiana described here. The results of this investigation showed the need to sensitize the rural population to rabies and the risks presented by vampire bats.

Communication efforts must be undertaken regularly for these populations and health professionals with messages that must be adapted and translated into the languages of the local occupants. Promotion of preventive measures should be encouraged, like the use of ample mosquito netting, blocking openings in the homes with screens, and perhaps the use of a light source during the night. The villagers should be encouraged to consult a doctor rapidly after a bat bite or scratch, regardless of the species.

Should a new episode of grouped cases in isolated regions or the increased frequency of exposures to bats, preventive anti-rabies vaccination for the entire populations of the concerned villages could be considered. This vaccination would avoid the RIG injections after bat bites and thus travel to the Cayenne CART. However, it requires the administration of 3 doses over 21–28 days, which can be difficult to program in Amerindian territories.

According to current regulations in French Guiana, it is not possible to allow RIG in an ARTO. Indeed, only a CART physician can administer them. Moreover, RIG are expensive and lose their efficacy if not maintained in the cold. Obviously, the risk of exposure to heat is higher in the isolated villages than in Cayenne. The common practice in France is to ensure safe conditions for the patient live including the capacity to be able to manage a shock syndrome in case of anaphylactic reaction. Therefore, when the CART doctors have to manage patients in remote areas, the presence of an emergency doctor with resuscitation material is safer but is often complicated to implement with the risk to delay the specific treatment in local conditions.

No veterinarian is located in the remote villages and, thus, the inhabitants were reminded to report the deaths or suspicious behavior of animals, particularly dogs, to the mayor. Domestic carnivores must be vaccinated against rabies. To facilitate the implementation of such a measure, a 3-year vaccination, as in Texas [39], could be envisaged for French Guiana for the most remote zones and performed by a mobile veterinary technician team as already performed for livestock.

## Supporting Information

Text S1 Dog population questionnaire.
(DOC)

Text S2 Regional Health Agency press release, 10 January, 2011.
(DOC)

Text S3 Prefecture of French Guiana Region communication, 11 January, 2011.
(DOC)
