# Supplementary material for: Rabies Risk: Difficulties Encountered during Management of Grouped Cases of Bat Bites in 2 Isolated Villages in French Guiana
Source: PLoS Negl Trop Dis. 2013 Jun 27;7(6):e2258. doi: 10.1371/journal.pntd.0002258 (PMC3694830; doi:10.1371/journal.pntd.0002258)
Supplement: Text S1 — Dog population questionnaire. (DOC) [file pntd.0002258.s001.doc]

**Dog population questionnaire**.

| **Interviewer**: …………………………………………………………….  Questionnaire completed on: ……………………. |
| --- |
| **Geographical information** 1) **Elaé** / **K**cayodé: …… |
| **Household information**  **2**) Owner: ……………………………………..  3) Is the property enclosed? (**F**ully, **P**artially, **N**o): ……  4) Type of household: **T**raditional house, **M**odern house, **O**ther: ……  5) Is the household occupied **P**ermanently or **O**ccasionally? : ……  6) Number of adults in the household: ……  7) Number of children in the household: ……  8) Do you know rabies? (**Y**es / **N**o): ……  9) In the last 5 years, did you hear about rabies cases in humans (**Y**es / **N**o: ……), how many cases?: ……. |
| **Dogs of the household**  10) Number of dogs, adult males: ……, adult females: ……,   juvenile males: …… juvenile females: ……, pups: ……  11) Who is in charge of:   |  | Children | Woman | Man | Elderly | | --- | --- | --- | --- | --- | | feeding dogs |  |  |  |  |   12) Is the dog routinely fed by people of the **H**ousehold, by **N**eighbours, **O**thers: ……  13) On what does the dogs feed?: lef**T**overs of the house, in **R**ubbish dumps, **O**ther: ……………..  14) Do neighbour's dogs enter regularly your property? (**Y**es / **N**o): ……  15) Do unknown dogs enter regularly your property? (**Y**es / **N**o): ……  16) Last year, how many of your dogs   |  |  | Pups | Juveniles | Adults | | --- | --- | --- | --- | --- | | 161 | died of disease |  |  |  | | 162 | died of accident |  |  |  | | 163 | disappeared |  |  |  | | 164 | have been abandoned |  |  |  | | 165 | have been killed by you*** |  |  |  | | 166 | have been culled during official campaign |  |  |  | | 167 | have been given or sold |  |  |  |   **** fill table in page 3 if dogs have been killed by the owner*  17) Who is mainly in contact with dogs?   |  |  | Children | Woman | Man | Elderly | | --- | --- | --- | --- | --- | --- | | 171 | Pups |  |  |  |  | | 172 | Juveniles |  |  |  |  | | 173 | Adults |  |  |  |  | |

| **Information on dogs, form 1** | | | | | |
| --- | --- | --- | --- | --- | --- |
|  | dog number | 1 | 2 | 3 | 4 |
| 18 | Sex (**M**ale, **F**emale, **G**estating female, l**A**ctating female) |  |  |  |  |
| 19 | For females,  date of last birth |  |  |  |  |
| 20 | number of cubs born |  |  |  |  |
| 21 | number of cubs still alive |  |  |  |  |
| 22 | number of cubs killed by the owner |  |  |  |  |
| 23 | number of litters since one year |  |  |  |  |
| 24 | number of cubs born since 1 year |  |  |  |  |
| 25 | race |  |  |  |  |
| 26 | Origin of the dog (i**N** house breeding, **G**ift, **P**urchase, **F**ound) |  |  |  |  |
| 27 | How old was the dog when it arrived? (years, months) |  |  |  |  |
| 28 | Since how long is it in the household? (years, months) |  |  |  |  |
| 29 | What is the role of the dog in the household? (garding **H**ousehold, **C**ompanion animal, hun**T**ing, **O**ther explain) |  |  |  |  |
| 30 | Is the dog tied up? ne**V**er, on **D**ay, on **N**ight, **A**ll day long |  |  |  |  |
| 31 | Does the dog stray? **S**ometimes, **O**ften |  |  |  |  |
| 32 | Can the dog be easily handeled? (**Y**es / **N**o) |  |  |  |  |
| 33 | Is the dog had been bitten by a vampire bat? (**Y**es / **N**o) |  |  |  |  |
| 34 | How many time during the last 12 months? |  |  |  |  |
| 35 | Is the dog vaccinated against rabies? (**Y**es / **N**o) |  |  |  |  |
| 36 | Is antirabies vaccination done during **c**ampaigns ? (**Y**es / **N**o) |  |  |  |  |
| 37 | Who does bring the dog for vaccination? (**C**hildren, **W**oman, **M**an, **E**lderly) |  |  |  |  |
| 38 | On which year was the last antirabies vaccination done? |  |  |  |  |
| 39 | Is a valid certificate for the antirabies vaccination available? (**Y**es / **N**o)***** |  |  |  |  |
| 40 | Notes |  |  |  |  |

| **Complement** |
| --- |

Why did owners kill some of their dogs since 1 year? (**F**emale cubs, **A**ggressive dog, **S**ick animal, to **R**educe the number of dogs, …)

|  | Cubs | Juveniles | Adults |
| --- | --- | --- | --- |
| 165-1 |  |  |  |
| 165-2 |  |  |  |
| 165-3 |  |  |  |
| 165-4 |  |  |  |
| 165-5 |  |  |  |
| 165-6 |  |  |  |
| 165-7 |  |  |  |
| 165-8 |  |  |  |
| 165-9 |  |  |  |
