# Supplementary material for: Rabies Risk: Difficulties Encountered during Management of Grouped Cases of Bat Bites in 2 Isolated Villages in French Guiana
Source: PLoS Negl Trop Dis. 2013 Jun 27;7(6):e2258. doi: 10.1371/journal.pntd.0002258 (PMC3694830; doi:10.1371/journal.pntd.0002258)
Supplement: Text S2 — Regional Health Agency press release, 10 January, 2011. (DOC) [file pntd.0002258.s002.doc]

**Regional Health Agency press release, 10 January, 2011.**

**RABIES: urgent mission of vaccination for 7 inhabitants of Elaé bitten by bats.**

THE FACTS

- Seven inhabitants of Elaé (Upper Maroni) living under the same lodging were bitten by bats during their sleep on the night of 24 December. This event was not reported by the victims and was discovered on Tuesday, 4 January by a doctor of Maripasoula during a routine visit.

- The victims, 5 of whom were under age 18, are doing well, but because the risk of rabies transmission by these bats cannot be excluded, the Pasteur institute of French Guiana, the Hospital of Cayenne and the Regional Health Agency organized an on-site mission of vaccination on Friday, 7 January. A new mission is planned in the next few days to complete the treatment.

- Usually, these treatments must be administered in a Centre for Anti-Rabies Treatment (CART). The need for rapid treatment, considering the delay since the bat bites, and the logistical difficulty of transporting all the victims to the Cayenne CART explain the exceptional deployment of a medical assistance mission to the isolated site of Elaé.

WHAT IS AT STAKE

- Rabies is a viral disease transmitted by the saliva of sick animals. Its symptoms appear on average 6 weeks after the bite or animal scratch, with a range of 1 week to several months.

- Rabies is always fatal once the symptoms appear. However, curative treatment by vaccination associated or not with specific immunoglobulin injections, is effective when administered before their appearance.

- Rabies is present in French Guiana; indeed a man died from rabies in 2008. In our region the rabies virus can be transmitted by all the bat species and warm-blooded, wild or domestic mammals, including dogs and cats.

- “Vampire” bat bites are painless. It is not rare that people sleeping and protected by a mosquito net are bitten during the night, but grouped cases of bat bites are very unusual.

HOW TO AVOID THE INFECTION?

Avoid contact with animals at risk of transmission (bat, unvaccinated dogs and cats):

- Protect your children: do not let them play with animals;

- Never handle a bat dead or alive;

- Avoid stray animals and inform the veterinarian services;

- Vaccinate your pets, since they can be bitten by a rabid bat and infect you with rabies through scratches or bites;

- Sleep under a mosquito net in non-closed spaces (shelters, hammocks in the forest);

- Block the openings in houses to avoid the intrusion of bats (wire nettings, boards).

In the case of an animal bite, you must immediately:

- Thoroughly wash the wound with water and soap, and then disinfect it with an antiseptic;

- Consult a doctor who will treat the wound, check your anti-tetanus vaccination, and organize the specific curative treatment in coordination with the CART.

- You can also call the Pasteur Institute of French Guiana CART for advice: 05 94 29 26 00
