# Supplementary material for: Rabies Risk: Difficulties Encountered during Management of Grouped Cases of Bat Bites in 2 Isolated Villages in French Guiana
Source: PLoS Negl Trop Dis. 2013 Jun 27;7(6):e2258. doi: 10.1371/journal.pntd.0002258 (PMC3694830; doi:10.1371/journal.pntd.0002258)
Supplement: Text S3 — Prefecture of French Guiana Region communication, 11 January, 2011. (DOC) [file pntd.0002258.s003.doc]

**Prefecture of French Guiana Region communication, 11 January, 2011.**

**The prefect of French Guiana region and the regional health agency communicate:**

**Recall of precautions to avoid the risk of rabies**

Seven inhabitants of Elaé (Upper Maroni) living under the same lodging were bitten by bats during their sleep on the night of 24 December. The victims, five of whom were under age 18 years, are doing well, but because the risk of transmission of rabies by these bats cannot be excluded, an on-site vaccination mission was organized on Friday, 7 January.

**How to avoid the infection ?**

Avoid contact with animals at risk of transmission (bat, unvaccinated dogs and cats):

- Protect your children: do not let them play with animals;

- Never handle a bat dead or alive;

- Avoid stray animals and inform the veterinarian services;

- Vaccinate your pets, since they can be bitten by a rabid bat and infect you with rabies through scratches or bites;

- Sleep under a mosquito net in non-closed spaces (shelters, hammocks in the forest)

- Block the openings in houses to avoid the intrusion of bats (wire nettings, boards)

**In case of an animal bite**, you must immediately:

- **Thoroughly wash** the wound with water and soap, and then disinfect it with an antiseptic.

- **Consult a doctor** who will treat the wound, check your anti-tetanus vaccination, and organize the specific curative treatment in coordination with the CART.

- You can also call the Pasteur Institute of French Guiana CART for advice: 05 94 29 26 00
